# Supplementary material for: Development and Validation of a Case-Based Survey Assessing Ethical Decision-Making in Prehospital Resuscitation
Source: Healthcare (Basel). 2025 Jan 30;13(3):267. doi: 10.3390/healthcare13030267 (PMC11817982; doi:10.3390/healthcare13030267)
Supplement: Supplementary file 1 [file healthcare-13-00267-s001.zip › Supplementary S3- Results from the exploratory factor analysis .pdf]

Supplementary S3: Results from the exploratory factor analysis

```
. sortl

Rotated factor loadings (pattern matrix) and unique variances sorted
```

Variable	Factor1	Factor2	Factor3	Factor4	Factor5	Uniqueness
pt_onske_p~p	0.8635	0.1435	0.0660	-0.0686	-0.0132	0.2245
ikke_dok_dnr	0.8570	0.1151	0.0762	-0.0573	-0.0536	0.2403
dok_dnr	0.6608	-0.2074	0.0342	0.2188	0.2450	0.4113
p_oenske	0.5762	0.0136	0.5004	-0.0537	-0.1144	0.4015
fysisk_til~d	0.5438	0.4154	-0.0351	0.0175	0.1331	0.5125
livskval_f~d	0.4756	0.3047	0.2446	0.1383	-0.3152	0.5026
erfaringer	0.0420	0.7726	0.0351	-0.0512	0.0526	0.3947
pt_livskval	0.1344	0.7526	0.0759	0.0982	0.0152	0.3999
subjektive~r	0.3944	0.3982	0.1926	0.0795	0.0661	0.6381
klage	-0.0397	-0.0043	0.8321	0.0372	0.1631	0.2781
p_reaktioner	0.1948	0.1164	0.8034	-0.0675	0.0030	0.2984
vaerdig	0.2282	0.1381	0.1179	-0.7312	0.1863	0.3456
pt_alder	0.2075	0.2994	0.0934	0.6688	0.1803	0.3788
belaegning	-0.0064	0.0609	0.1081	-0.0225	0.8826	0.2050
