# Supplementary material for: Development and Validation of a Case-Based Survey Assessing Ethical Decision-Making in Prehospital Resuscitation
Source: Healthcare (Basel). 2025 Jan 30;13(3):267. doi: 10.3390/healthcare13030267 (PMC11817982; doi:10.3390/healthcare13030267)
Supplement: Supplementary file 1 [file healthcare-13-00267-s001.zip › Supplementary S2- Modified Delphi process ΓÇô Round 1.pdf]

Supplementary S2: Modified Delphi process – Round 1

**Consideration**	**No. of experts (n=8) ranking the consideration as important**
The right of the patient to a dignified end of life	7
Patient age	6
The patient's wishes regarding resuscitation attempts if this gets expressed verbally by the health care staff	6
The patient's expected quality of life if successfully resuscitated	6
Patient's physical condition	5
The patient's wishes regarding resuscitation attempts if a written document exists, e.g. a printed living will	5
The patient's wishes regarding resuscitation attempts if this gets expressed verbally by the patient's relatives	5
The relatives' emotional reactions	5
The risk of getting a complaint	5
Your own safety (e.g. threats from bystanders)	5
The patient's social status	4
Your subjective assessment of the patient's quality of life up to the cardiac arrest	4
The importance of the patient's physical surroundings (e.g. their home)	4
Helping the relatives to understand the resuscitation is futile	3
Your experiences from previous cardiac arrests	3
Complying with guidelines	3
Any knowledge you have on the occupancy situation at the intensive care units in the reception area	3
The patient's religious or cultural background	2
The patient's ethnicity	2
The expressed wish of relatives about resuscitation	2
Disagreements with other healthcare professionals about resuscitation (e.g. ambulance personnel)	2

Best possible distribution of prehospital resources (e.g. it takes time and resources from the ambulance that could be used to treat another patient, if you choose to initiate treatment)	2
Best possible distribution of in-hospital resources (e.g. it takes time and resources from a large in-hospital team if you choose to transport the patient)	2
Your own ethical and cultural values	1
Your wish on resuscitation if you were the patient	1
Your wish on resuscitation if your relatives were the patient	1
Respecting the efforts of the non-medical personnel before your arrival	1
Initiating resuscitation to use the opportunity to train resuscitation e.g. own learning or education of colleagues	1
The relatives' perceived expectations	0
