# Supplementary material for: Development and Validation of a Case-Based Survey Assessing Ethical Decision-Making in Prehospital Resuscitation
Source: Healthcare (Basel). 2025 Jan 30;13(3):267. doi: 10.3390/healthcare13030267 (PMC11817982; doi:10.3390/healthcare13030267)
Supplement: Supplementary file 1 [file healthcare-13-00267-s001.zip › Supplementary S1- The final questionnaire to describe the ethical decision making process in EMS.pdf]

Supplementary S1: The final questionnaire to describe the ethical decision making process in EMS

Questionnaire study of Danish emergency physicians' consideration of ethical factors in decision-making for pre-hospital resuscitation of adults

SECTION 1
**<u>Scenarios involving resuscitation</u>**<br>**This section presents five sample scenarios involving resuscitation attempts. Please assess whether or not you would attempt resuscitation**
A 65-year-old man collapses in his own home. His wife witnesses the incident but does not initiate CPR. The ambulance has a 5-minute travel time, while the emergency doctor's van has a 10-minute travel time. Upon arrival, paramedics initiate a resuscitation attempt and rhythm analysis. When you arrive, the paramedics have been performing CPR for 5 minutes. The patient's initial heart rhythm is PEA.<br><br>What would you choose to do here?<br><br>☐ Continue the resuscitation attempt<br>☐ End the resuscitation attempt
Why would you choose to end the resuscitation attempt here?
Why would you choose to continue the resuscitation attempt here?
A 64-year-old woman collapses in her own home. She is found by her spouse, who heard a thud coming from the living room. He immediately calls 112 but does not initiate CPR. The ambulance has a 5-minute travel time, while the emergency doctor's van has a 10-minute travel time. The patient's initial heart rhythm is PEA. When you arrive at their home, you find a document clearly stating that the patient does not want resuscitation. The patient's spouse is also at home and insists, "the document should be disregarded and everything be done to save my wife."<br><br>What would you choose to do here?<br><br>☐ Continue the resuscitation attempt<br>☐ End the resuscitation attempt
Why would you choose to end the resuscitation attempt here?
Why would you choose to continue the resuscitation attempt here?
A 65-year-old woman collapses in her own home. The nursing staff who have been attending to the patient because of her multiple sclerosis have had telephone contact with the patient 5 minutes earlier and have now found her lifeless at her home. They initiate CPR. The emergency doctor's van arrives 5 minutes later at the same time as the ambulance. While rhythm analysis is being performed, the nursing staff say that the patient has an extremely poor quality of life and rarely goes outside her door. The first rhythm is VF.

What would you choose to do here?<br><br>☐ Continue the resuscitation attempt<br>☐ End the resuscitation attempt
Why would you choose to end the resuscitation attempt here?
Why would you choose to continue the resuscitation attempt here?
A 63-year-old man collapses in his camper at a camping site. He is seen collapsing by an employee at the place, but due to some confusion, CPR is not initiated. The emergency doctor's van has a 10-minute travel time. You arrive at the same time as the ambulance. The camping site employee explains that the man "is probably addicted to all kinds of stuff." The home is messy and dirty, with several liquor bottles lying around. You observe that the patient is cachectic and has caput medusae. When the paramedics place the pads, the first rhythm is PEA.<br><br>What would you choose to do here?<br><br>☐ Continue the resuscitation attempt<br>☐ End the resuscitation attempt
Why would you choose to end the resuscitation attempt here?
Why would you choose to continue the resuscitation attempt here?
A 65-year-old woman collapses in her own home. This is witnessed by the patient's entire family (about 15 people). They call 112, but CPR is not initiated due to language barriers. You arrive at the patient's home after 7 minutes at the same time as the paramedics. There are many people in the apartment and several of them are shouting and crying. The paramedics attach pads and find PEA. Due to language barriers, it is difficult to get any more information from the family. The atmosphere in the apartment is becoming increasingly chaotic.<br><br>What would you choose to do here?<br><br>☐ Continue the resuscitation attempt<br>☐ End the resuscitation attempt
Why would you choose to end the resuscitation attempt here?
Why would you choose to continue the resuscitation attempt here?

SECTION 2							
**Ethical considerations in pre-hospital resuscitation attempts:<br>Please rate the importance of the factors below on a scale ranging from "extremely unimportant" to "extremely important."<br><br>Please base your rating on the majority of your experienced pre-hospital cardiac arrests.**							
	Extremely unimportant	Moderately unimportant	A little unimportant	Neither important nor unimportant	A little important	Moderately important	Extremely important
The patient's age							
The patient's physical condition prior to the cardiac arrest							
Your subjective assessment of the patient's quality of life up to the cardiac arrest							
The patient's perception of the patient's own quality of life prior to the cardiac arrest, **e.g. as expressed by health care staff or relatives**							
The patient's expected quality of life if successfully resuscitated							
The patient's wishes regarding resuscitation attempts if a written document exists, **e.g. a printed living will**							
The patient's wishes regarding resuscitation attempts if this gets expressed verbally by the healthcare staff							
The patient's wishes regarding resuscitation attempts if this gets expressed verbally by the patient's relatives							
The right of the patient to a dignified end of life							
The expressed with of relatives about resuscitation							
The relatives' emotional reactions							
The risk of getting a complaint							
Your experiences from previous cardiac arrests							
Any knowledge you have on the occupancy situation at the intensive care units in the reception area							
Do you have any other ethical considerations in your decision to attempt resuscitation that are not mentioned above?							

If so, which:

SECTION 3	
**Reliable sources of information on the patient and the situation**	
Tick the three sources of information that matter the most to you<br>(Multiple choice)	☐ Healthcare staff<br>☐ Relatives<br>☐ Medical record information in print or electronically<br>☐ Information from the patient e.g. written note<br>☐ Own observations of the situation<br>☐ Verbal information from the patient's own doctor<br>☐ Bystanders

SECTION 4	
**Documentation of your decision-making in pre-hospital resuscitation attempts**	
Do you document any ethical consideration that you included in your decision to attempt resuscitation in the patient's medical record?<br>(Single choice)	☐ Each time<br>☐ Most times<br>☐ Sometimes<br>☐ Seldom<br>☐ Never
What is the reason that you do not always document any ethical considerations when attempting resuscitation?<br>Please tick all applicable response options	☐ Lack of time<br>☐ I don't think it is relevant<br>☐ Lack of instructions<br>☐ Other
Note down any other reasons:	

SECTION 5	
**Inclusion of ethical considerations in decision-making for resuscitation**	
Would a guideline be useful that included ethical factors in connection with prehospital resuscitation attempts?<br>Ethical factors refer to e.g. the patient's quality of life, the patient's wishes, the relatives' attitudes, or your own values<br>(Single choice)	☐ Yes, it would be useful<br>☐ No, I don't think it would be used<br>☐ Don't know

Do you think that a guideline of ethical factors would make the decision on resuscitation easier?<br>(single choice)	☐ Yes<br>☐ No<br>☐ Don't know
Do you have sufficient knowledge on ethical factors during a resuscitation attempt to be able to include it in your decision?<br>(single choice)	☐ I have the necessary knowledge to include ethical factors in my decision<br>☐ I don't think it is relevant<br>☐ I lack knowledge on ethical factors<br>☐ I do not feel equipped to include ethical factors in my decision
Is there a need for more guidance concerning ethical factors in resuscitation attempts?	☐ Yes<br>☐ No, it is sufficient<br>☐ I don't think it is relevant

SECTION 6	
**Background information**	
Age	
Gender	☐ Male<br>☐ Female<br>☐ Other
What is your primary clinical work area	☐ Intensive<br>☐ Palliative<br>☐ Clinical anesthesia<br>☐ Pain management<br>☐ Prehospital<br>☐ Other
What other primary work area?	
Which region do you work as a prehospital emergency doctor?	☐ Region of Southern Denmark<br>☐ Central Region<br>☐ North Region<br>☐ Capital Region<br>☐ Region Zealand
How many years have you worked as a prehospital emergency doctor?	
